# The role of innovation for economy and sustainability of photovoltaic modules

**DOI:** 10.1016/j.isci.2022.105208

**Published:** 2022-09-27

**Authors:** Ian Marius Peters, Jens A. Hauch, Christoph J. Brabec

**Affiliations:** 1 Forschunsgzentrum Jülich, Helmholtz Institute Erlangen-Nürnberg for Renewable Energy HI ERN, Immerwahrstraße 2, 91058 Erlangen, Germany; 2 Institute Materials for Electronics and Energy Technology (i-MEET) Friedrich–Alexander University Erlangen–Nürnberg (FAU), 91058 Erlangen, Germany

**Keywords:** Engineering, Energy management

## Abstract

The role of innovation for the success of photovoltaics cannot be overstated. Photovoltaics have enjoyed the most substantial price learning of any energy technology. Innovation affects photovoltaic performance in more ways, though. Here, we explore the role of innovation for economics and greenhouse gas savings of photovoltaic modules using replacement scenarios. We find that the greenhouse gas displacement potential of photovoltaic modules has improved substantially over the last 20 years—4-fold for the presented example. We show that the economically ideal time for repowering is after around 20 years, but that repowering may reduce greenhouse gas savings. Expanding photovoltaic installations is generally preferable, economically and sustainably, to repowering. We argue that i) we should maximize the greenhouse gas saving potential of each module, which requires a global strategy, ii) tandem solar cells should aim for stability, and iii) efforts to continue and accelerate innovation in photovoltaic technology are needed.

## Highlights

• Innovation influences the economy and sustainability of solar panels significantly
• Innovation has increased the ability of solar panels to displace carbon 4-fold
• Installations should maximize the carbon displacement potential of each panel
• Capacity expansion should be prioritized over system replacements

## Graphical abstract

**Figure:**
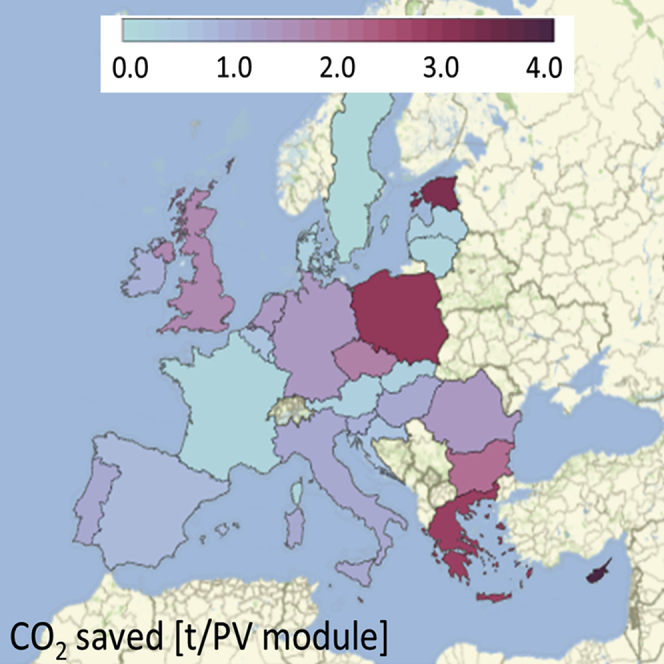

## Introduction

**Figure 1:**
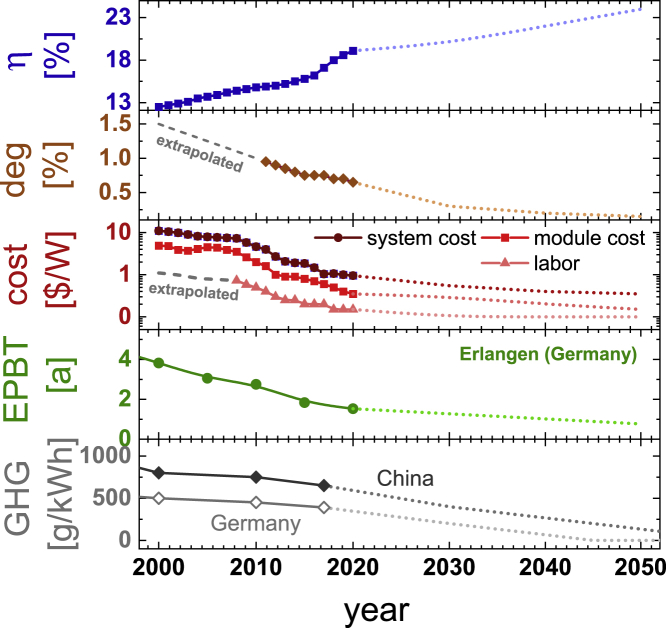
Development of the most relevant technical, economic, and sustainability parameters of PV modules over time Data for module efficiency (η), degradation rate (deg), and cost until 2020 were taken from (ISE, 2020; Bermudez and Perez-Rodriguez, 2018; Fu et al., 2018; Feldman et al., 2012; SETO, 2017; Leccisi et al., 2016), future projections until 2050 are based on (IEA, 2020; BMU, 2021; Li et al., 2020) or were extrapolated were data was not available (marked gray). Energy payback time values were calculated using data from (Bermudez and Perez-Rodriguez, 2018; Fu et al., 2018; Feldman et al., 2012; SETO, 2017; Leccisi et al., 2016; IEA, 2020; BMU, 2021; Li et al., 2020) and the greenhouse gas emissions associated with electricity generation in Germany and China was taken from (Bloomberg, 2021; UN, 2020).

Innovation in performance and manufacturing has propelled photovoltaic (PV) technology from the exception to the norm. The manifestations of innovation are defined as improvements in key technical, economic, and sustainability parameters pertaining to PV modules. The price learning of solar electricity is without precedent in energy technology, with a reduction of more than 99% in the last 40 years (IRENA 2019). A report by the European REFLEX project (Louwen et al., 2018), for example, mentions learning rates of 18.6 ± 1% for PV systems, of 10.3 ± 3.3% for offshore wind systems, and of 5.9 ± 1.3% for onshore wind systems. PV modules have steadily become cheaper, more efficient, and more reliable, and they will continue to do so (ISE, 2020; Bermudez and Perez-Rodriguez, 2018; Green 2005; Fu et al., 2018, Feldman et al.,. 2012; SETO, 2017; ISE, 2015; van Beuzekom et al., 2018; Peters et al., 2021). The rates of progress in improving the key metrics module efficiency *η*, degradation rate, and system costs are essential for past economic successes and the prospects of photovoltaics. Moreover, improvements in energy payback time (EPBT) of photovoltaic modules (Bermudez and Perez-Rodriguez, 2018; Fu et al., 2018; Feldman et al., 2012; SETO, 2017; Leccisi et al., 2016; IEA, 2020a, 2020b; BMU, 2021; Li et al., 2020) and reductions in the global warming potential of the energy mix in PV module producing- and installing countries (Bloomberg, 2021; UN, 2020) affect key sustainability metrics like the ability of a PV module to displace greenhouse gases (GHG), on which we focus in this publication. Past developments and future projections for these metrics are shown in Figure 1.

In this paper, we explore how the rate of progress in photovoltaic technology affects economic decisions in PV system planning, the introduction of disruptive technologies, and the GHG saving potential of PV modules. Our tool of choice for this exploration is the replacement scenario. In a replacement scenario, a photovoltaic module installed in year 1 is replaced with an improved module in year 2, and the response of a target metric is observed. In economic observations, the target metric is revenue. The dynamics of revenue optimization are tied to the rate of progress—rapid innovation make replacements attractive, though future improvements may make it worthwhile to wait longer. Tandem solar cells are a special case of innovation in photovoltaics with the prospect of boosting conversion efficiency further than conventional solar cells can. Module replacement has been suggested as a viable option for market introduction (Jean et al., 2019) for tandems, and we explore this premise in the context of projected, conventional innovation.

When exploring sustainability, we adhere to a definition of the term given in the “Lexikon der Nachhaltigkeit”, which can be summarized as: “Sustainability aims at the long-term protection of tangible/intangible goods and/or economic/ecological units.”(Aachener Stiftung, 2015). The goal of solar panels, as we see it, is the protection of the prevailing favorable climatic conditions by transitioning toward a carbon-free energy production. As such, solar panels contribute to sustainability development goal number 7, “affordable and clean energy” as formulated by the United Nations Development program (UNDP, 2015). In this context, the target metric we use is GHG emission savings. The dynamics of these savings are similar to those in economics, with one important difference: the aspired quick reduction in the GHG emission of energy mixes around the world gives a strong preference to systems being installed earlier. Note that our study is limited to this particular aspect of sustainability, and we do not discuss impacts on other sustainability goals like material conservation through recycling (Farrell et al., 2020).

Replacement scenarios provide insights on multiple levels: They have immediate relevance for economic considerations in the context of PV repowering (Fregosi et al., 2020) and, as mentioned, the market introduction of novel technologies (Jean et al., 2019). Beyond that, we use replacement scenarios as an analysis tool to quantify the impact of innovation on the economics and sustainability of PV installations and to illustrate the rate of innovation over time. We find that innovation in photovoltaics has created benefits that go beyond the widely noticed price learning (IRENA, 2019), and has, for example, substantially improved our ability to use solar panels to address climate change.

### Economic considerations for replacing photovoltaic modules

#### Module replacement with and without innovation

**Figure 2:**
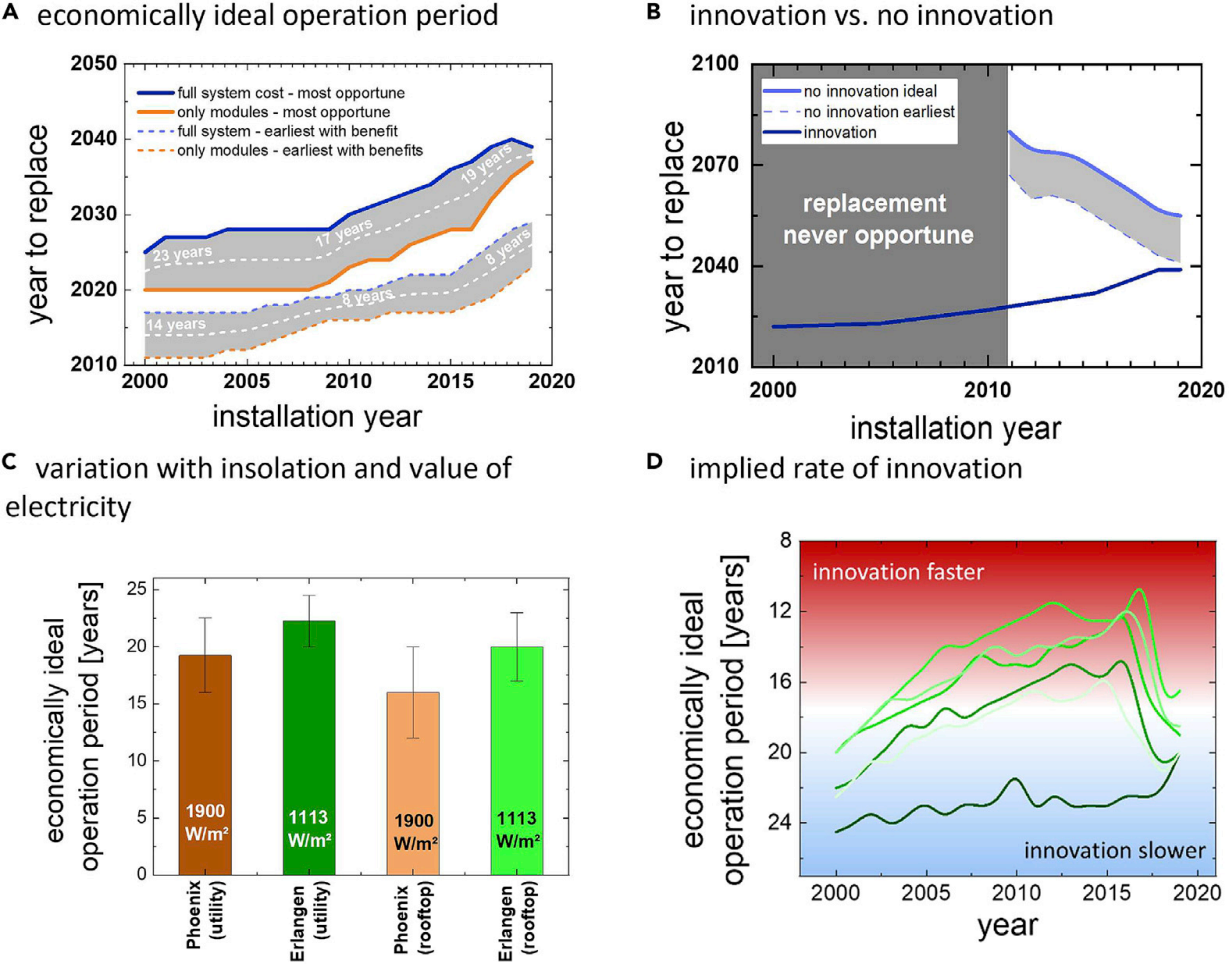
Innovation and economic performance (A) economically ideal operation period. Ideal- (thick, solid lines) and earliest (thin segmented lines) year to replace modules are displayed as a function of installation year. Shown are two scenarios, one in which only modules are replaced (orange), and one in which the entire installation is replaced (blue). (B) innovation vs. no innovation. The graph compares the ideal year to replace for two scenarios,one in which innovation is considered and one in which it is neglected. (C) variation with insolation and value of electricity. The figure shows how the economically ideal operation period varies with insolation and value of electricity. The given range corresponds to replacing only modules (lower end) and the entire system (upper end). (D) implied rate of innovation. The figure depicts the ideal operation period as a function of time for several scenarios with variations in insolation and electricity value. We propose that this metric could be used to determine the pace of innovation as it comprises all relevant techno-economic factors, with a shorter time until replacement indicating a higher pace of innovation.

The rates at which the techno-economic characteristics of PV modules improve are relevant for determining when it is economically beneficial to replace an existing PV module with a new one. To determine at what point module replacement becomes beneficial, we calculate the convergence value of the net present value ($NPV_{\infty }$) of a PV installation in which modules are replaced in a certain year (see van Beuzekom et al., 2018 for additional features of this approach). Installed and replaced modules have an efficiency, degradation rate, and incur cost depending on the year of installations and with values given in Figure 1. All systems and models follow NREL’s System Advisor Model (Blair et al., 2018), and status reports from NREL (Fu et al., 2018). Replacement is considered beneficial, as soon as $NPV_{\infty }$ is greater if modules are replaced than if they are not. Results are shown in Figure 2A and were calculated for a rooftop installation in Erlangen with a discount rate of 6.9% and a value of electricity of 0.3€/kWh. We show two sets of results there, one labeled “most opportune” and marked as thick solid lines, and one labeled “earliest with benefit”, and marked with thin segmented lines. The most opportune scenario corresponds to maximizing $NPV_{\infty }$, whereas earliest with benefits marks the break-even point between replacing and not replacing modules. In each set, we calculate two scenarios, one in which only modules are replaced—i.e. replacement costs include module costs and installation labor, labeled “only modules”, and colored in orange, and one in which the entire installation is replaced, labeled “full system costs”, and colored in blue. Costs for module disposal and temporary loss of revenue are neglected. These scenarios can be seen as a best- and worst-case scenario in terms of cost, and the truth will likely be somewhere in between. We find that the economically ideal operation period for this scenario in the year 2000 was 23 years. The ideal operation period reduces to 17 years for modules installed in 2010 and slightly increased again to 19 years for modules installed around 2020. The earliest time after which replacement becomes opportune was 14 years for modules installed in 2000 and around 8 years in 2020. These variations reflect the past and projected pace of improvements in techno-economic module performance. Sensitivity analysis reveals that cost reductions and efficiency improvements share a roughly similar impact on this duration, whereas reductions in degradation rate had a smaller impact.

How strongly innovation affects the choice for when to replace solar panels is shown in Figure 2B. Here, we compare the calculated most opportune time for module replacement in scenarios with and without innovation. No innovation signifies that module efficiency, degradation rate, and cost remain the same as they were at the moment of installation. Generally, a lack or under-appreciation of the rate of innovation will result in an overestimation of the ideal operation time. Without innovation, there would never have been an advantage of replacing modules installed in or before 2011 (note that we included no incentives in this calculation). By how much the ideal period of operation is overestimated reduces over time, yet even for modules installed today, neglecting innovation results in overestimating the economically ideal operation period by more than 15 years.

The economically ideal operation period depends on insolation as well as on the value of electricity. This finding is shown in Figure 2C, where we compare this period for Erlangen, Germany (1113 kWh/m^2^) and Phoenix, US (1900 kWh/m^2^) (Global Solar Atlas, 2022) for a rooftop installation with a value of electricity of 30 ct/kWh and a utility installation with a value of electricity of 10 ct/kWh. Greater insolation and greater value of electricity both reduce the optimum operation period.

The duration before replacement can be used as a metric for the overall rate of innovation as it comprises all techno-economic factors shown in Figure 1. We show the calculated ideal operation period as a function of year of installation for a number of scenarios with different insolation (1113 kWh/m^2^ and 1900 kWh/m^2^) and values of electricity (10, 20, and 30 ct/kWh) in Figure 2D. Defined like this, the pace of innovation is a function of time, and in most scenarios, the rate of innovation steadily increases from 2000 until between 2015 and 2017, and quickly reduces afterward. As results in later years dominantly depend on projected improvements, the reduced pace of innovation is indicative of projections being conservative.

Some insights into the rationale behind efficiency projections can be gained from comparing different sources—for other parameters, significantly fewer consistent projections were available. Projections differ in terms of the absolute module efficiency that is considered attainable, and the speed with which improvements become available. A major distinction for the absolute value is whether a study assumes that tandem technology will enter the mainstream market or not. Studies shown in Figure 1 are skeptical about the prospect of tandems and converge at around 24% efficiency in 2050. Studies that assume tandems will become mainstream, like for example Goldschmidt et al. (2022) do, state higher numbers in the range of 27% in 2050 and 35% in 2100. Based on efficiency alone, such a development would indicate a faster pace of future innovation. The reason why these projections were not included was that a consistent cost projection for tandem technology is missing. Tandems are fundamentally more expensive than single junction solar cells from the same technology (Peters et al., 2016). These higher costs offset the pace of innovation at least partially. Regarding the pace of innovation, compared to projections used by PV ICE (Ovaitt et al., 2022), we use a more constant rate of improvement, in line with the scenario for PERC cells in ITRPV (ITRPV, 2021) for example, whereas PV ICE uses an initial fast pace that slows down after around 2025. Consequently, the PV ICE projections result in a pace of innovation that slows down later and more rapidly.

Whether these conservative projections anticipate a regression in learning from currently very high rates, as for example seen in price learning, or whether they reflect an under-appreciation of the potential of the technology, as frequently seen in projections for installations (Creutzig et al., 2017), only the future can tell.

### Sustainability considerations for replacing photovoltaic modules

#### The greenhouse gas savings model

To calculate the impact of a photovoltaic module on greenhouse gas emissions (Jäger-Waldau et al., 2020), we propose a model that balances emissions during module production and savings during operation. GHG are generated mostly due to the energy required during module production. Embodied carbon (Raugei et al., 2017) in a PV module depends on the GHG emissions of the electricity mix of the location where the module is produced, and the module production’s energy demand. Note that we use the energy mix of a given country, China and Germany in the examples. The rationale for this choice is to reproduce the larger energy context. We are aware that this choice can be challenged and comment on it in the discussion section. In this study, we calculated embodied carbon $L_{CO2,eq}$ in terms of an equivalent amount of CO_2_ from the energy payback time EPBT and the GHG emissions GHG of the location of production (see Figure 1):

(Equation 1) $$L_{CO2,eq}\left(t_{0},x\right)=EPBT\left(t_{0}\right)\cdot \;\frac{I\left(x\right)}{1000}\cdot \eta \left(t_{0}\right)\cdot A\cdot GHG\left(t_{0}\right)$$

In this equation, I(x) is the specific yield in kWh/kWp in a given location, Erlangen in the example shown below, $\eta \left(t_{0}\right)$ is the efficiency of the used PV panel in the year of installation $t_{0}$, and A is the unit area of a PV module (here 1.7 m^2^). We chose this indirect way because we found more consistent references for these values than for the development of embodied carbon over time directly. The obtained values follow those published by Leccisi et al., 2016—the calculation enables tracking changes over time. Because countries reduce their GHG emissions and because module production has become more energy efficient, embodied carbon reduces over time.

After installation, we assume that the module displaces an amount of electricity that would otherwise have been generated with the energy mix of the country in which the module is installed. The module itself produces a negligible amount of GHG during operation; hence, an amount of GHG equal to that produced by the energy mix is saved. This assumption could exemplarily be imagined as a house that, after installation of PV panels, becomes energetically autonomous and is disconnected from the grid. Because of continuing decarbonization, the potential to save further GHG emissions diminishes over time. Once a country reaches carbon neutrality, electricity generation with the PV module no longer saves any additional greenhouse gasses. This approach takes into account a given baseline of decarbonization in the energy mix. This decarbonization is, at least partially, achieved through the installation of PV modules. The method presented here can be considered a perturbation approach; a small change to the baseline—the installation of a single or a small number of modules—is studied. The overall capacity to be installed until 2045 is not affected.

Energy yield (EY) of a PV installations is calculated using a simple degradation model

(Equation 2) $$EY\left(t,x\right)=I\left(x\right)\cdot \eta \left(t_{0}\right)\cdot \text{A}\cdot {\left(1-\text{deg}\left(t_{0}\right)\right)}^{t}$$

In this equation, $\text{deg}\left(t_{0}\right)$ is the annual degradation rate of the module. The amount of GHG generation saved by a PV-module $TB_{CO2}$ was calculated via:

(Equation 3) $$TB_{CO2,eq}\left(t,x\right)=\;EY\left(t,x\right)\cdot GHG\left(t\right)$$

Finally, the cumulative GHG savings $CB_{CO2,eq}$ are obtained by summing up the savings each year and subtracting the amount of embodied carbon.

(Equation 4) $$CB_{CO2,eq}\left(t,x\right)=-L_{CO2,eq}\left(t_{0},x\right)+\sum_{t}EY\left(t,x\right)\cdot GHG\left(t\right)$$

#### Carbon saving of a PV panel

**Figure 3:**
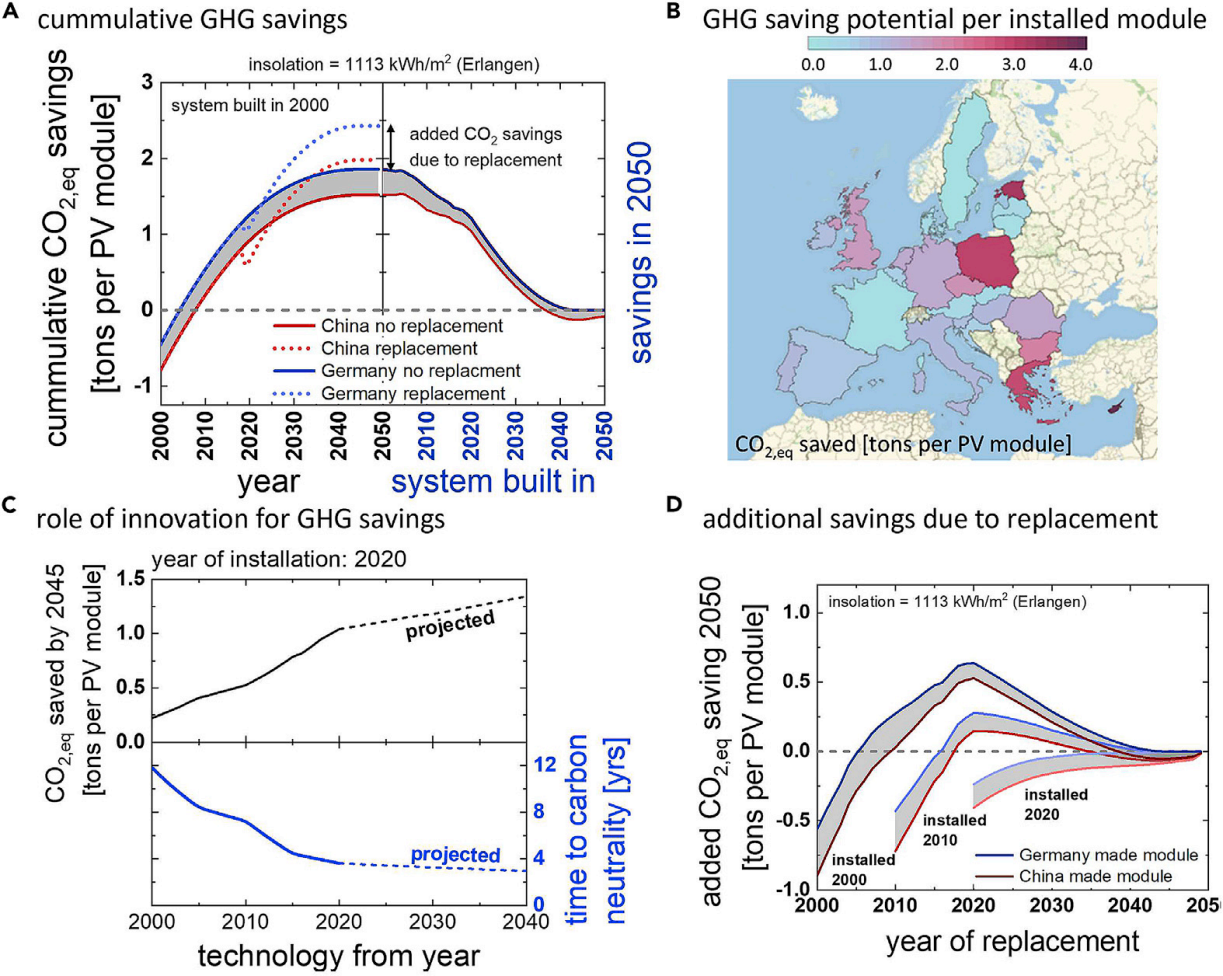
Innovation and sustainability (A) cummulative GHG savings per module in a PV system. The figure illustrates how GHG savings accumulate for a module produced in China (red) and Germany (blue) until 2050 for a module installed in the year 2000 in Erlangen, Germany (left). Dotted lines indicate savings if the module is replaced with state of the art in the year 2020. The right side shows savings until 2050 as a function of installation year. (B) GHG saving potential per installed module. (The calculated saving potential for a module installed in 2020 and operated until 2050 for countries in the EU and the UK. Data were taken from the European Environment Agency, 2022 are shown. (C) role of innovation for GHG savings. The figure shows the influence of innovation on GHG savings. The hypothetical scenarios shown here assume a China-made module with techno-economic features from a given year (Figure 1) which was installed in 2020 and operated until 2045 in Erlangen. (D) additional savings due to replacement. The figure shows the benefits and detriments on GHG savings of replacing modules with state of the art ones.

In a first exercise, we calculated the cumulative carbon saving in units of t_CO2,eq_ that a module installed in Erlangen would generate. Note that we focus here only on the module and that there is additional carbon embodied in the remaining system that would add embodied carbon for any components that is replaced. Figure 3A (left) shows how a module installed in the year 2000 over time accumulates CO_2,eq_ savings. Due to the embodied carbon, it takes a few years for the module to obtain a positive CO_2,eq_ balance. How long this takes depends on where the module was produced; a module produced in Germany breaks-even in its fifth year of operation, a module produced in China in its eighth. Savings converge in 2045, when Germany plans to become carbon neutral. Note that this does not mean that we can stop installing PV modules after 2045. New electricity demand will have to be balanced by new sources, which need to be net carbon-free to maintain carbon neutrality. Our results just mean that there will be no *additional* GHG savings. The right hand side of Figure 3A shows how the cumulative savings in 2045 depend on when the module is installed. Even though efficiencies increase over time (see Figure 1), the ambitious goals for decarbonization and the shorter operation time until 2045 result in later installed modules having a smaller carbon saving potential. According to current projections, modules produced in China after 2037 could even be said to have an overall negative effect and result in more GHG emissions if installed in Germany due to imported embodied carbon. Though some caution is needed with this argument (see embodied carbon discussion below). Note that this examination neglects additional emissions due to transportation (Hu et al., 2017).

Figure 3B shows how the carbon saving potential of a photovoltaic module installed in the year 2000 for countries in the European Union and the UK. In the calculation shown here, we have adopted the common, European goal of decarbonization by 2050 for every country, even if goals for individual countries differ. The saving potential primarily depends on the GHG emissions of each country. Installing photovoltaic panels in countries with high carbon intensities like Poland, Cyprus, or Greece would be most effective in reducing GHG emissions. Installations in countries like Denmark or Norway, on the other hand, would make little contribution and could even hurt the overall carbon balance through imports of embodied carbon.

In Figure 3C, we explore the significance of innovation for GHG savings. The scenario shown here uses a module installed in the year 2000 but with techno-economic features from the year shown on the x axis (see Figure 1). Technological features for the year 2000 include an efficiency of 12.5% and an EPBT (1700W/m^2^) of 2.5 years. Installed in 2020, such a module would by 2045 have displaced 220 kg of CO_2,eq_ and would have taken more than 12 years to displace the same amount of GHG as was needed for its production. A state-of-the-art module from 2020 with 19.1% efficiency and 1 year EPBT (1700W/m^2^) will by 2045 have displaced more than 1000 kg of CO_2,eq_ and would need less than 4 years to become GHG positive. If the projected technology of 2040 had been available already in 2020, these values would change to more than 1300 kg and less than 3 years. Improving the techno-economic features has a significant impact on the contribution to sustainability that each photovoltaic module can make. Sensitivity analysis reveals that efficiency improvements and the corresponding reduction in EPBT had the strongest impact on improving GHG savings, followed by reductions in GHG emissions, and a small contribution from improvements in degradation rate.

An additional finding that was indicated in Figure 3A is that module replacement can improve GHG savings (dotted lines). Replacing modules that were originally installed in 2000 with new and better modules in 2020 improves total saving in 2045 by more than 30%. This situation marks a best-case scenario, though, as can be seen in Figure 3D. This figure shows the additional savings due to module replacement as a function of when replacement takes place and for three different years of original installation (2000, 2010, and 2020). The later modules are installed the smaller the benefit from module replacements becomes, and the earlier modules should be replaced to maximize benefits. For modules installed after 2017, replacement always either leaves the balance equal or results in greater CO_2_ emissions. The nearby conclusion, to not replace modules, could be seen though as being in conflict with economic recommendations (see Figure 2).

To be able to calculate the introduced metrics, a time series for all parameters needs to be assumed. Used parameters were summarized in Figure 1.

#### Tandem solar cells

A promising current innovation is the development of perovskite-based tandem solar cells. Tandems are expected to push PV module efficiencies to above 30% without concentration. As higher module efficiencies improve the economic balance, particularly in the long-term, module replacements are an attractive market introduction scenario for tandems. One such scenario for perovskite on silicon tandem technology was investigated by Jean et al., in 2018. The study concluded that replacement was beneficial and that higher efficiencies would allow using technologies with a higher degradation rate and still be profitable.

**Figure 4:**
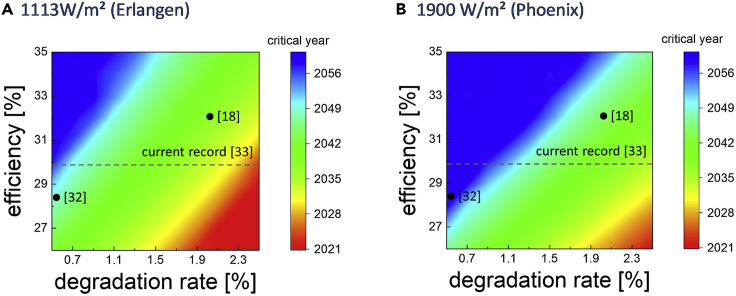
Innovation and tandem solar cells Year until which a tandem with given efficiency, degradation rate, and a cost of 33 ct/W will have an advantage compared to single junction technology developing according to Figure 1 in a replacement scenario (termed “critical year”). Figure (A) on the left shows a utility installation in Erlangen with 1113 W/m^2^ insolation. Figure (B) on the right shows an installation in Phoenix with 1900 W/m^2^.

Tandem technology is not immediately available and will, hence, compete with future single junction technologies rather than current ones. To explore the economic advantage of tandems, we model until when a tandem with a certain techno-economic performance retains an advantage over single junction technology when replacing modules. For this purpose, we calculate until which year the $NPV_{\infty }$ for a tandem with an efficiency between 25% and 35%, a degradation rate between 0.5% and 2.5%, and a module cost of 33 ct/W is greater than that of a single junction with performance according to Figure 1. The year in which a tandem with given properties loses its economic advantage over state-of-the-art single junctions solar cells is termed “critical year”. Performance values for tandems are oriented on values published in Jean et al., 2019 and Sofia et al., 2018. We calculated scenarios for utility installations in Erlangen and Phoenix with a value of electricity of 10 ct/W. Results are shown in Figure 4.

Degradation rate and efficiency are both decisive for the competitiveness of tandem technology. Whether and for how long a given tandem retains an advantage over single junction technology depends also on where it is deployed; greater insolation benefits tandems. We investigated a number of different published scenarios in this context. Jean et al., 2019 discuss a variety of values, arguing that a tandem with high efficiency could stay competitive, even if degradation exceeds that of current technology. One scenario mentioned in this publication is a tandem with 32% efficiency and a degradation rate of 2%. We find that such a tandem would outperform single junction technology in a replacement scenario in both Erlangen and Phoenix, though the tandem would retain its advantage longer in Phoenix (until 2044) than in Erlangen (until 2037).

How the trade-off between efficiency and stability plays out can be seen in comparison with another scenario. In a publication by Sofia et al., 2018, a tandem is discussed with 28.4% efficiency and annual degradation on-par with state-of-the-art silicon solar cells—0.5%. Such a tandem would retain an advantage in Phoenix until 2056 and in Erlangen until 2048. This finding emphasizes the strong lever of improving degradation rates on tandem competitiveness, a result that can also be seen when considering the current world record for a perovskite-silicon tandem of more than 31% (CSEM, 2022). For this very recent result, no efficiency data are available. For the previous record device, 95% performance after 300 hours was published (Al-Ashouri et al., 2020), which cannot be translated into a meaningful value for long-term performance. Depending on the degradation rate, such a tandem would have no advantage even over state-of-the-art single junction solar cells today, if degradation rates are beyond 2.5%, or could remain superior until the 2060s, if rates are below 0.5%.

## Discussion and conclusions

### The value of innovation

The importance of innovation for the economic success and the ability to address climate change with photovoltaic technology cannot be overstated. Without innovation, the efficiency of solar panels would not have progressed so quickly and costs would not have come down so fast. Innovation has turned photovoltaic electricity production from a dream of idealists to the cheapest source of electricity ever available to mankind (IEA, 2020 II) in less than 40 years. Innovation has quadrupled the ability of a photovoltaic panel to displace GHG in a mere 20 years and will continue to increase it. Innovation is the motor that drives the fight against climate change. We find it worrying, consequently, that there are signs of a reducing pace in our ability to make solar panels even better and even cheaper. Figure 2D shows that the economically ideal operation period has been going down since 2017, indicating that techno-economic performance is not improving as fast as before. We also observe a reduced pace in improvements of GHG saving abilities after 2020 (Figure 3C). This observation is based on projections, and projections have consistently underestimated the innovative vim of the PV community in the past (Creutzig et al., 2017). Research and development for photovoltaic technologies is as important today as it was 20 years ago. Renewable energies are still at an early stage of their growth. Continued innovation will be essential to reach the ambitious installation goals required to achieve decarbonization.

### Replacing vs. building more

**Figure 5:**
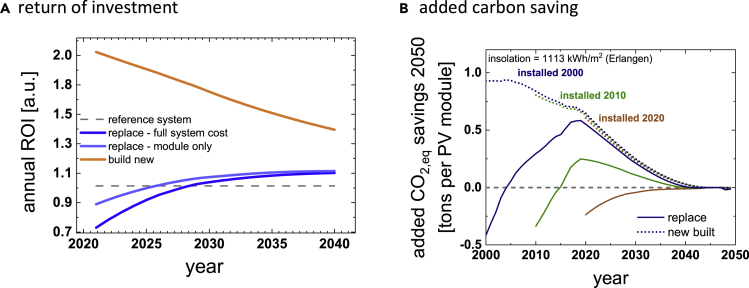
Replacement vs capacity expansions Comparison of replacement scenarios and expansion scenarios for the normalized economic return of investment (A) and the added carbon savings (B). Relative ROI was calculated by dividing the total ROI for the “replace” and the “build new” scenario by the ROI of the reference system without replacement or capacity addition. GHG savings were calculated similar to the results shown in Figure 3D (curves of that figure were used for the replacement scenario). Capacity expansion is always superior to replacement.

We use replacement scenarios as a tool to explore the impact of innovation on economics and sustainability of photovoltaic installations. Replacement scenarios are relevant in the context of PV-repowering (Fregosi et al., 2020) and as a strategy for market entry for new technologies (Jean et al., 2019). Economic considerations shown in Figure 2A indicate that module replacement is economically beneficial after about twenty years in many regions. For owners of rooftop installations, replacing old modules by state-of-the-art ones after this period could be a serious consideration and the same is true for utility installations at the end of a 25 year lifetime. This conclusion is problematic, though, for two reasons:

i) While replacement may be economically beneficial, it may result in a reduction of sustainability benefits. This issue was shown in Figure 3D. While for old modules, replacement is beneficial both in terms of economics as in sustainability, replacing more recently installed modules in the future will still incur an economic benefit but will reduce the carbon savings potential. For systems installed in Germany after 2017, module replacement increases GHG emissions. This reduction is due to a shift in the balance between embedded carbon and carbon displacement. From a standpoint of GHG savings, module replacement, for example through repowering, should be discouraged. This is not to say that repowering does not have its place. Repowering of installations that are retired, for example because they are defective or they are operating at a loss, is legitimate and makes sense.
ii) Module replacement neglects a better alternative: from an economic as well as from a sustainability standpoint, the benefits of installing additional modules outweigh those of replacement. This point is shown in Figure 5, in which we explore the economic implications of repowering versus building new in terms of return on investment ROI (left) and in terms of added CO_2_ savings per installed module (right). In either case, expanding capacity is the superior strategy.

The more general conclusion must be that, whenever possible, a PV module should be used for capacity expansion rather than for capacity improvement. This argument has a further deployment relevant component: reaching capacities of several tens of TW_P_ will require a massive expansion of module production capacities, material mining, and supply chain. This will not be an easy task. Module replacement reduces the capacity expansion potential of a new module compared to using it for an addition installations. The priority for the next several decades and until we are certain we can reach decarbonization goals must be to maximize the carbon saving potential of every newly produced module.

### Embodied carbon

Excessive anthropogenic GHG emission is a global problem and requires a global solution. The installation of PV modules is a strong indirect lever on GHG emissions—the installation of a PV module does not reduce GHG emission by itself, but it enables the displacement of power plants that burn fossil fuels. It therefore matters where a photovoltaic module is installed. An effective strategy to leverage the displacement potential of PV modules is to identify where the most carbon-intensive energy production sits, and install PV modules there. This strategy is effective because it front loads decarbonization and maximizes GHG savings. To achieve extensive decarbonization, though, all relevant GHG sources have to be replaced.

Similarly, it matters where a photovoltaic module is produced. The major contributor to embodied carbon in a PV module comes from the energy needed for its production, and the GHG intensity of this energy must be accounted for. The question now becomes which boundaries for energy production should we consider? A manufacturer could install solar panels to supply all energy needed for production and claim that his product (a PV module or anything else) now comes without embodied carbon. We would argue that this is a good course of action and should be encouraged because it avoids the construction of additional power plants that produce GHG, but that it does not qualify a claim of no embodied carbon. The rationale for our argument is that the manufacturer is not isolated from its surroundings. They depend on an infrastructure of suppliers and workers that create a large and intertwined root system.

The next larger boundary that could be considered is the local power grid because it supplies entire communities with electricity. Many countries, including Germany, the US, and China, have several grids that are interconnected to different degrees. Using one wide-area synchronous grid is a meaningful choice of unit, especially if grid operation is largely independent, as is the case in North America. Germany and China have grids that are more closely interconnected and are more centrally regulated. Our choice to use GHG emission values per country was motivated in parts by considering the administrative power over electricity supply, which is predominantly on the national level. The other part of the motivation was that decarbonization goals are under national jurisdiction. If nations decide how to decarbonize, they also bear responsibilities for GHG produced within their boundaries.

An alternative choice to countries is economic regions. This approach was, for example, followed by Fraunhofer ISE (Bett, 2022), and is motivated by the argument that regions like the European Union have joint goals and tightly interconnected infrastructure. These include interconnected electricity grids that blur the relation between where something is produced and where the electricity for the production is coming from. We find this choice meaningful, yet we would argue that also within the European Union, renewable energies should predominantly be installed in countries with high levels of GHG emission. For the results presented here, it makes little difference whether figures from the EU or Germany are used, as, while Germany is above the EU average, it is close.

Finally, one could argue that GHG emissions should be considered only globally. We would respond that there is no globally active entity with the power to enforce action. While it is in our best interest to act jointly and globally, the execution of measures to control global warming is the responsibility of nations or federations with joint powers and responsibilities. What consequences does this have for embodied carbon and the import of solar panels? The issue we mentioned in context with Figure 3A is that if Germany fulfills its decarbonization plan, after 2037 importing PV modules from China would result in an increase in GHG emissions globally, because the overall reduction potential in Germany falls below the embodied carbon for modules produced in China. That does not mean, however, that Germany should necessarily stop importing PV modules from China or other countries with levels of GHG emission. Without continued PV installation after 2037, Germany will likely not be able to achieve the goal of complete decarbonization. Embodied carbon can be handled by either taking it into account in the decarbonization goals or by reducing it, for example by installing locally produced PV modules. Considering that the GHG reduction potential of a PV module is realized by enabling decommissioning of combustion power plants, one could also argue that there are better places to install a PV module than Germany in 2037.

So, what are the recommendations? First, decarbonization strategies need to develop from national into global strategies. Initially, it is necessary for countries to focus on their individual energy transition and deploy the necessary infrastructure. Though, after initial steps are taken, a global strategy for where to install new renewable energy source is needed to maximize their potential. Second, nations need to share responsibilities for embodied carbon on exports and imports. If a country like Germany wants to decarbonize quickly, it cannot rely on manufacturing in areas with higher levels of GHG emission. Global supply chains should follow GHG emission upstream not downstream. Outsourcing production into countries with high GHG emissions can reduce national values, but will increase GHG emission globally. Nations bear the responsibility of their energy mix, but they also bear the responsibility of the imported embodied carbon. This aspects needs to be considered for GHG goals, also.

### Tandems need stability

Tandem technology is one of the most innovative current developments in photovoltaics, and market entry for tandems could benefit from repowering. A replacement scenario was introduced by Jean et al. with regard to perovskite-silicon tandem solar cells. One conclusion from this analysis was that higher efficiencies could enable market entry for tandems with greater than state-of-the-art degradation rates. In principle, we can confirm the findings of this study, but we have a few caveats. First: stability, represented as low degradation rates, is a very strong lever on economic competitiveness (van Beuzekom et al., 2018). A tandem that matches state-of-the-art degradation can have significantly lower efficiency and will still outperform a high-efficiency, high-degradation tandem. In a competition between tandems, the one with greater stability will likely be the winner. Second: tandems are a future technology and compete with future single junction solar cells. Low degradation rates will help retain a competitive advantage for tandems longer than high degradation rates and high efficiency. Third: perovskite-silicon tandems are already competitive in terms of efficiency, but are nowhere near that goal in terms of stability. To date, there are no published results for perovskite solar cells that would allow a direct comparison with silicon on long-term degradation. While there are promising results that indicate that perovskites can have long-term stability (Prasanna et al., 2019), efficiency records take the spotlight in research and publications. The focus on efficiency is unwarranted as a stable 20% solar cell can easily outperform an unstable 30% solar cell.

### Limitations of the study

Prediction is very difficult, especially if it is about the future. Numeric conclusions of this study are based on projections of the future developments of photovoltaic technology development and carbon reduction goals. Both should be taken with a grain of salt. We have attempted to describe how faster or slower developments affect our findings; still, the assumption is that development will continue somewhat similarly to how it happened in the past. Disruptive technological changes both from inside the photovoltaic industry (a replacement of silicon technology by perovskites), or from outside (the availability of cheap fusion energy) would change the picture entirely.

## STAR★Methods

### Key resources table

**Table:** 

REAGENT or RESOURCE	SOURCE	IDENTIFIER
**Software and algorithms**		

OriginPro 2020	OriginLab	https://www.originlab.com/index.aspx?go=Products/Origin
Wolfram Mathematica 12.0.0.0	Wolfram	https://www.wolfram.com/mathematica/?source=nav
Global Solar Atlas	Solargis	https://globalsolaratlas.info/map?c=11.609193,8.4375,3
System Advisor Model 2018.11.11	NREL	https://sam.nrel.gov/

**Other**		

2018. U.S. Solar Photovoltaic System Cost Benchmark: Q1 2018	NREL/TP-6A20-72399	https://www.nrel.gov/docs/fy19osti/72399.pdf.
Photovoltaic (PV) Pricing Trends: Historical, Recent, and Near-Term Projections	DOE/GO-102012-3839	https://www.nrel.gov/docs/fy13osti/56776.pdf
Current and Future Cost of Photovoltaics. Long-term Scenarios for Market Development, System Prices and LCOE of Utility-Scale PV Systems	Fraunhofer ISE / AGORA Energiewende	https://www.ise.fraunhofer.de/content/dam/ise/de/documents/publications/studies/AgoraEnergiewende_Current_and_Future_Cost_of_PV_Feb2015_web.pdf.
Greenhouse gas emission intensity of electricity generation in Europe	European Environment Agency	https://www.eea.europa.eu/ims/greenhouse-gas-emission-intensity-of-1

### Resource availability

#### Lead contact

Further information and requests for resources and reagents should be directed to and will be fulfilled by the lead contact, Ian Marius Peters (im.peters@fz-juelich.de).

#### Material availability

This study did not generate new unique reagents.

### Experimental model and subject details

This study does not use experimental methods typical in the life sciences.

### Method details

#### Modelling

All mentioned Mathematica scripts are made available as supplemental information item “Data S1”. Results in Figure 2 were calculated according to the methods described in Peters et al. 2021. The calculations can be carried out by using numbers from Figure 1 and the System Advisor Model from NREL. A Mathematica script entitled “value of maintenance” was used to automate the process and calculate time series to speed up the process. Results shown in Figure 3 were calculated using Equations 1, 2, 3, and 4, implemented in Mathematica with scripts entitled “Sustainability” and “CO2 savings in Europe”. Data for GHG emissions in Europe was taken from the European Environment Agency. Results shown in Figure 5 were calculated using Mathematica with the script entitled “tandem”. Results in Figure 5 were calculated using the scripts “value of maintenance” and “sustainability”.

## Data Availability

This paper analyzes existing, publicly available data. Information about access to the used datasets are listed in the key resource table and are given in the reference list. This paper does not report original code. All calculations can be carried out using the mentioned software or the given equation. All Mathematica scripts have been submitted as supplemental information. Any additional information required to reanalyze the data reported in this paper is available from the lead author upon request.

## Supplemental information

Data S1. Mathematica Files used in this document File “202012 value of maintenance steady state replacement and new buil.nd” was used to calculate results shown in Figures 2 and 5. File “202103 Sustainability.nb” was used to calculate results shown in Figure 4. File “202111 CO2 Savings.nb” was used to calculate results shown in Figure 4. File “202205 tandem.nb” was used to calculate results shown in Figure 3.
